# Supplementary figures and images for: Development of an Innovative Online Dietary Assessment Tool for France: Adaptation of myfood24
Source: Nutrients. 2022 Jun 28;14(13):2681. doi: 10.3390/nu14132681 (PMC9268261; doi:10.3390/nu14132681)

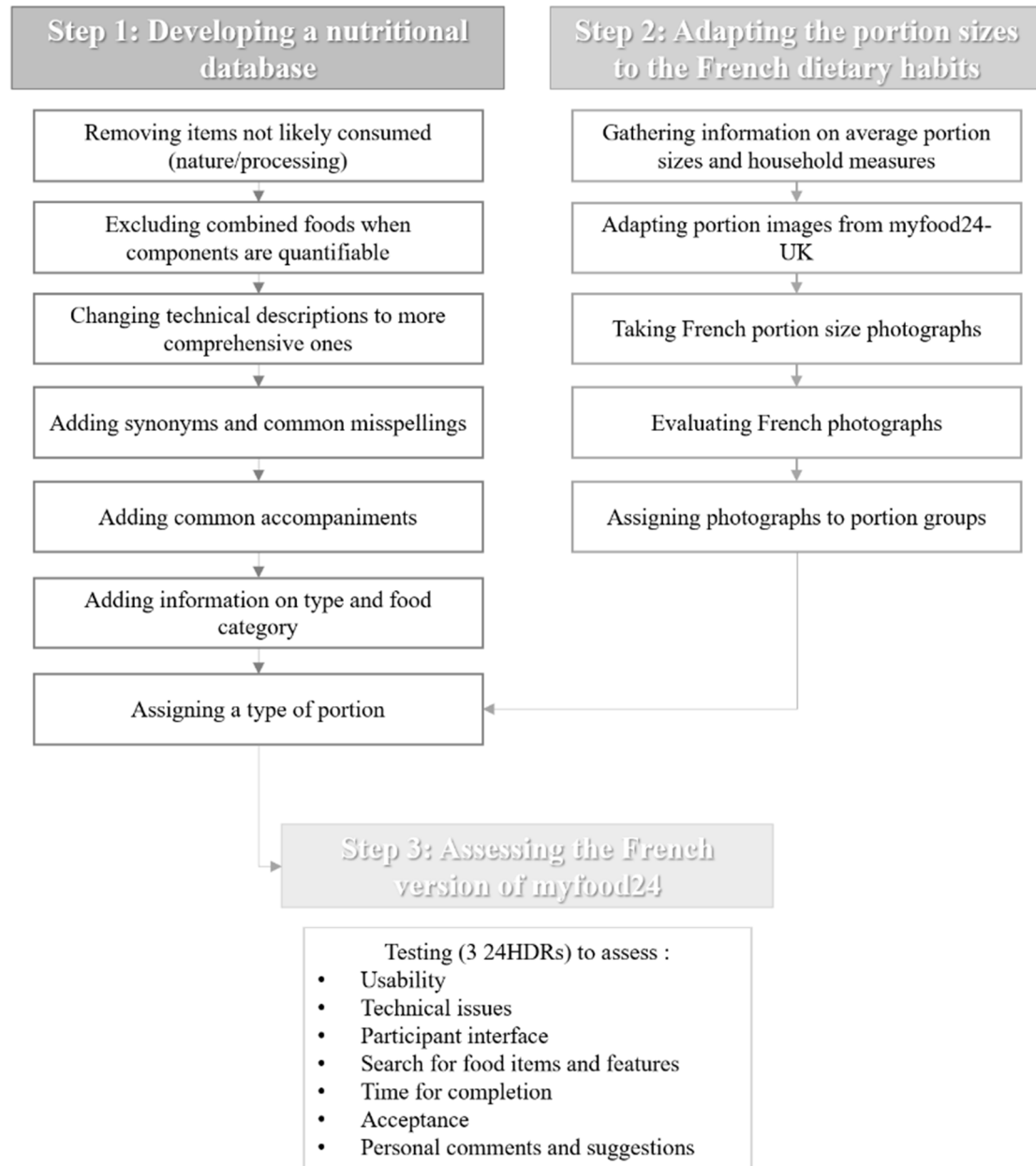

### Overview of the applied methodology of myfood24 France's development

Supplement: Supplementary file 1 [file nutrients-14-02681-s001.zip › nutrients-1753899-supplementary.pdf]
